# Fibrinogen and Albumin Score Changes during Preoperative Treatment Can Predict Prognosis in Patients with Locally Advanced Rectal Cancer

**DOI:** 10.1155/2019/3514586

**Published:** 2019-11-13

**Authors:** Meng-Jun Tang, Shu-Bo Ding, Wang-Yuan Hu

**Affiliations:** Department of Radiation Oncology, Jinhua Municipal Central Hospital, Jinhua, China

## Abstract

**Background:**

Fibrinogen (Fib) and albumin (Alb) levels are indicators of systemic inflammatory responses. Elevated Fib and decreased Alb levels are considered negative prognostic factors in different types of cancer. Here, we explored the prognostic value of changes in pre- and post- neoadjuvant chemoradiotherapy (NCRT) plasma fibrinogen and serum albumin (FA) scores in patients with locally advanced rectal cancer (LARC).

**Methods:**

A total of 106 patients with LARC who underwent NCRT followed by surgical resection at Jinhua Municipal Central Hospital between 2011 and 2015 were analyzed. In addition, plasma Fib and serum Alb levels before and after NCRT were collected. FA scores were calculated based on the Fib and Alb levels dichotomized by clinical reference values. Patients were classified into two groups based on the changes in FA scores during NCRT: in group A, FA scores decreased or remained unchanged (*n* = 84), and in group B, FA scores increased (*n* = 22). Changes in FA scores were compared with patient outcomes.

**Results:**

Increased FA scores were associated with worse disease-free survival (DFS) and overall survival (OS) in patients with LARC. The occurrence of systemic failure was higher in group B than in group A (40.9% vs. 19%, *P* = 0.032). In multivariate analysis, changes in FA scores, pretreatment carcinoembryonic antigen (CEA) levels, and pathologic differentiation were independent prognostic parameters for DFS and changes in FA scores and pretreatment CEA levels were independent prognostic parameters for OS.

**Conclusions:**

Increased FA score after NCRT was an independent negative prognostic factor for DFS and OS in patients with NCRT-treated LARC.

## 1. Introduction

In 2018, colorectal cancer accounted for over 1.8 million newly diagnosed cases and 881,000 cancer-related deaths globally [1]. In China, the morbidity and mortality associated with colorectal cancer have increased in the last decade [2]. Approximately 60% patients with rectal cancer are initially diagnosed with locally advanced tumors with regional and/or distant metastasis. Despite rapid improvements in multimodality therapies, the 5-year disease-free survival (DFS) rate in such patients is still unsatisfactory [3, 4]; distant metastasis is the most prominent cause of treatment failure. Therefore, it is imperative to explore promising indicators that can identify locally advanced rectal cancer (LARC) with high risk of recurrence and unfavorable outcome.

Recently, accumulating evidence has suggested that the prognosis in patients with malignancy depends not only on tumor characteristics but also on host factors, including the level of inflammation and immune and nutritional status [5–8]. Moreover, combinations of inflammation-related index systems and nutritional parameters, such as the C-reactive protein/albumin (Alb) ratio (CAR), defined as the ratio of C-reactive protein to Alb, and the prognostic nutritional index (PNI), calculated based on Alb concentration and lymphocyte count, have been reported as useful predictors in various types of cancer [9, 10]. Fibrinogen (Fib) and Alb are inflammatory markers and are considered useful prognostic parameters in several types of cancer. High levels of circulating Fib have been reported to correlate with unfavorable outcomes in esophageal cancer, lung cancer, and colon cancer [11–13]. Moreover, hypoalbuminemia has been associated with adverse outcomes in various malignancies [14, 15]. Recently, the combination of Fib and Alb levels, which can reflect coagulation activity, systemic inflammation, and nutritional status, was proposed as a useful prognostic marker in various malignancies [16–18].

However, the prognostic significance of changes in Fib and Alb (FA) scores during neoadjuvant chemoradiotherapy (NCRT) in patients with LARC remains unclear. Therefore, this study aimed at evaluating the prognostic value of changes in FA scores during NCRT in patients with LARC.

## 2. Methods

Patients diagnosed with rectal cancer (clinical stage II/III) who underwent NCRT and radical surgery with a curative intent at Jinhua Municipal Central Hospital from January 2011 to December 2015 were reviewed. Of them, patients with hematological, hepatic, or kidney diseases; autoimmune diseases; infections; or other malignancies and those with incomplete data or lost to follow-up were excluded. In total, 106 patients who met the inclusion criteria were included and analyzed. This single-center, retrospective study was approved by the Ethics Committee of Jinhua Municipal Central Hospital.

The initial clinical stage was assessed by total colonoscopy and pelvic magnetic resonance imaging. All patients were concurrently treated with long-course radiation therapy consisted of 45 Gy in 25 fractions over 5 weeks and oral administration of capecitabine (1650 mg/m^2^/d) on days 1–14 and 22–35. Total mesorectal excision (TME) surgery was performed 5–11 weeks after the completion of chemoradiotherapy (CRT). All patients were recommended for adjuvant chemotherapy, starting at approximately 3–6 weeks after surgery. Six patients (5.7%) did not receive adjuvant chemotherapy due to poor physical condition or rejection, 68 patients (64.2%) were treated with 4–6 cycles of oxaliplatin and capecitabine (XELOX) every 3 weeks after surgery, and 32 patients (30.2%) were treated with 4–6 cycles of single capecitabine.

Follow-up was performed every 3 months for the first 2 years and every 6 months for the following 3 years. Locoregional recurrence was defined as any recurrence involving anastomosis and lymphatic vessels and adjacent organs in the pelvic cavity. Distant metastasis was considered as any tumor spread outside the pelvic cavity. DFS was calculated from the first day of NCRT to disease progression (either locoregional recurrence or distant metastasis) or last follow-up. Overall survival (OS) was calculated from the first day of NCRT to the time of death or last follow-up. All patients were followed up to death or until March 2019.

All patients' clinicopathological characteristics were collected, including age, sex, tumor distance from anal verge, pathological differentiation, TNM stage, pathological examination, and adjuvant therapies. Postoperative tumor pathological TNM (ypTNM) stage was established using the TNM classification system recommended by the American Joint Committee on Cancer/International Union Against Cancer Colorectal Cancer TNM Staging System (7th edition). Tumor downstaging was defined as ypT0~2N0M0 after surgery. Pathologic complete remission (pCR) was defined as ypT0N0M0.

Blood samples were examined within 1 week before CRT (pre-CRT) and at 2–4 weeks after CRT (post-CRT). Carcinoembryonic antigen (CEA), carbohydrate antigen 19-9 (CA19-9), Fib, and Alb levels were all measured. Serum CEA levels > 5 ng/ml and CA19‐9 levels > 37 ng/ml were considered abnormal. The FA score was determined based on plasma Fib and serum Alb levels. According to our hospital reference, serum Fib levels were dichotomized into normal (≤4 mg/dl) and high (>4 mg/dl). Alb levels were classified into two categories as low (≤35 g/l) and normal (>35 g/l). Patients with high Fib and low Alb levels scored 2. Those with high Fib and normal Alb levels or normal Fib and low Alb levels scored 1, and those with normal Fib and normal Alb levels scored 0.

## 3. Statistical Analyses

All statistical analyses were performed using the Statistical Package for Social Sciences, version 19.0 (SPSS Inc., Chicago, IL, USA). Intergroup differences in clinicopathological features were analyzed by the chi-square and Fisher's exact tests. Differences in continuous variables were analyzed using Student's *t*-test. Survival curves were plotted using the Kaplan–Meier method, and survival rates were compared using the log-rank test. Potential prognostic factors for survival outcomes were identified in multivariable analysis using a Cox proportional-hazards model. *P* < 0.05 was considered statistically significant.

## 4. Results

### 4.1. Patient Characteristics and Pathological Stages

The clinicopathological characteristics of patients included in the study are presented in Table 1. Of the 106 patients enrolled, 74 (69.8%) were men, and the median age was 60 years (range 19–83 years). Clinical stage II and III cancers were diagnosed in 45 (42.5%) and 61 (57.5%) patients, respectively. Histologic examination revealed adenocarcinoma in 101 patients. All patients received concurrent chemotherapy and radiotherapy. The median distance from tumor to anal verge was 5 cm (range 2–12 cm). The median time from completion of NCRT to surgery was 58 days (range 40–77 days).

After NCRT and radical resection, we examined pathologic staging. The numbers of patients with ypT0, T1, T2, T3, and T4 were 24 (22.6%), 5 (4.7%), 46 (43.4%), 29 (27.4%), and 2 (1.9%), respectively. Pathological lymph node metastases were observed in 26 patients (24.5%). The number of patients downstaged to yp stage I or below was 63 (59.4%). The number of patients with pCR was 23 (21.7%).

### 4.2. Correlation between Changes in FA Scores and Clinicopathological Factors

The number of patients with FA scores of 0, 1, and 2 before treatment was 85, 16, and 5, respectively. The number of patients with FA scores of 0, 1, and 2 after NCRT was 75, 24, and 7, respectively. Patients were classified into 2 groups: in group A, post-CRT FA scores were decreased or unchanged compared with pre-CRT FA scores, and in group B, post-CRT FA scores increased compared with pre-CRT FA scores. Corresponding patient characteristics are presented in Table 2. Patients in group B were more frequently elderly individuals (*P* = 0.017), had higher post-CRT Fib levels (*P* ≤ 0.001), and had lower post-CRT Alb levels (*P* = 0.003). No significant differences were observed between the two groups for other factors, such as sex, tumor distance from anal verge, histology, CEA or CA19-9 levels, time to surgery, pathologic T stage, pathologic N stage, lymph vascular invasion (LVI), and perineural invasion (PNI).

### 4.3. Association between Changes in FA Scores and Outcomes of Patients with LARC

**Figure 1:**
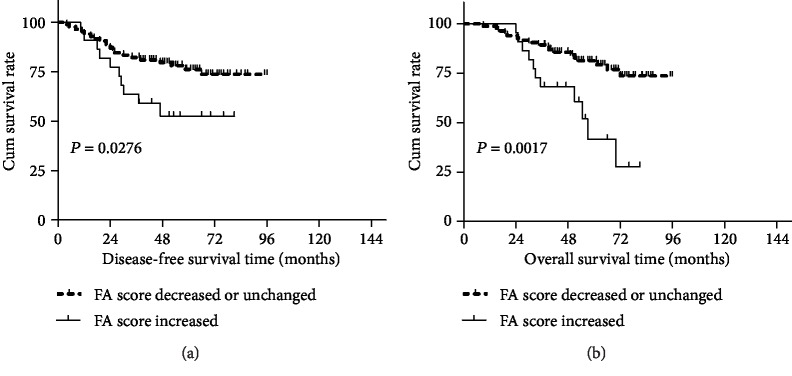
Correlation between changes in FA scores and prognosis of LARC patients. Patients in the increased FA score group exhibited a significantly worse disease-free survival (a) and overall survival (b).

The median follow-up period for all patients was 42 months (range 8–95 months). There were 30 cases of recurrence and 29 deaths up to the last follow-up. The estimated 3-year DFS and 3-year OS rates were 78.3% and 84.9%, respectively, for all patients. Patients with increased FA scores had lower 3-year DFS rate than those with decreased or unchanged FA scores (63.6% vs. 82.1%, *P* = 0.028; Figure 1(a)); similarly, the 3-year OS rate was significantly lower in patients with increased FA scores than those with decreased or unchanged FA scores (68.2% vs. 89.3%, *P* = 0.002; Figure 1(b)).

The 3-year locoregional recurrence-free survival (LRFS) was 93.6% for the whole cohort. The 3-year LRFS was not significantly different between group A and group B (94.6% vs. 90.0%, *P* = 0.254). The 3-year distant metastasis-free survival (DMFS) was 81.0% for all patients. The 3-year DMFS rate was significantly higher in group A than group B (84.5% vs. 67.2%, *P* = 0.022).

Of the 106 patients, local recurrence occurred in 10 patients; 5 (4.7%) patients presented with only locoregional recurrence, and 5 presented with both locoregional recurrence and distant metastases. Only distant metastases were observed in 20 (18.9%) patients. In group A, 16 patients (19%) presented with distant metastases (liver in 5 patients, lung in 5 patients, bone in 1 patient, retroperitoneal lymph node in 1 patient, peritoneal dissemination in 1 patient, anastomosis and liver in 2 patient, and pelvic and retroperitoneal lymph node in 1 patient). In group B, 9 patients (40.9%) presented with distant metastases during follow-up (liver in 2 patients, lung in 3 patients, peritoneal dissemination in 2 patients, pelvic cavity and lung in 1 patient, and inguinal node and pelvic lymph node in 1 patient).

### 4.4. Uni- and Multivariate Analyses of DFS and OS

In univariate analyses (Table 3), pathologic differentiation, CEA and CA19-9 levels, pCR, pathologic N stage, downstaging, and changes in FA scores were associated with DFS and OS.

In multivariate COX regression analysis (Table 4), pathologic differentiation (HR 3.922, 95% CI 1.519-10.125, *P* = 0.005), pretreatment CEA levels (HR 5.741, 95% CI 2.407-13.690, *P* ≤ 0.001), and changes in FA scores (HR 2.433, 95% CI 1.102-5.375, *P* = 0.028) were independent prognostic parameters of DFS. Pretreatment CEA levels (HR 3.832, 95% CI 1.646-8.924, *P* = 0.002) and changes in FA scores (HR 3.325, 95% CI 1.468-7.532, *P* = 0.002) were independent prognostic parameters of OS.

## 5. Discussion

The findings of the present study can be summarized as follows: (1) Increased FA scores after CRT were associated with high risk of postoperative systemic failure in patients with LARC. (2) Changes in FA scores were an independent prognostic parameter for patients with LARC treated with NCRT and radical surgery. To the best of our knowledge, this is the first study to evaluate the prognostic impact of FA score changes in patients with LARC.

Plasma Fib is an important coagulation-related protein involved in the maintenance of hemostasis. Fib is also an acute phase protein involved in inflammatory responses. Several inflammatory cytokines, such as interleukin 6 (IL-6) and IL-1, have been known to regulate Fib synthesis [19, 20]. An increasing number of studies have shown that plasma Fib has utility as a strong predictor of malignancy, and high levels of plasma Fib are significantly correlated with unfavorable outcomes in several cancers, including ovarian and pancreatic cancer [21–23]. Furthermore, Tang et al. noted that elevated plasma Fib levels were associated with advanced tumor stage and adverse prognosis in colorectal cancer [24]. However, the molecular mechanisms underlying the association between Fib levels and tumor progression have not been comprehensively elucidated. Fib regulates tumor cell proliferation, migration, angiogenesis, and metastasis by directly binding to members of the transforming growth factor b (TGF-b), vascular endothelial growth factor (VEGF), fibroblast growth factor (FGF), and platelet-derived growth factor (PDGF) families [25]. Fib can inhibit the recognition of tumor cells from immune cells by stabilizing the tumor cell-platelet adhesion conjugate, leading to evasion of monitoring by the host immune system and facilitating the metastasis of tumor cells [26].

Serum Alb is commonly served as a nutritional parameter. McMillan et al. demonstrated that the most important reason for the decreased Alb concentration in patients with cancer is not disorder in Alb synthesis or increases in transcapillary leakage rates but the accelerated degradation of Alb, secondary to systemic inflammatory responses to the host [27]. Proinflammatory cytokines released by tumor tissue and related inflammatory cells, such as IL-6 and IL-4, can affect Alb synthesis in hepatocytes, leading to reduction of Alb levels [28]. Therefore, apart from reflecting nutritional status, Alb levels can implicate inflammatory response as well as Fib levels. The clinical effect of hypoalbuminemia on rectal cancer has also been investigated. Cengiz et al. showed that low Alb levels were related to large tumor sizes and distance metastases [29]. In a study of 431 patients with colorectal cancer undergoing curative surgery, serum Alb level was identified as a reliable prognostic marker for survival, with a 25% increase in the risk of death for each 5 g/l reduction in serum Alb levels [30]. However, most of the previously mentioned studies focused on high plasma Fib levels or low serum Alb levels at the time of pretreatment, with dreadful prognosis in patients with rectal cancer, without taking into consideration of changes during the preoperative treatment. Therefore, we performed the present study to assess the clinical significance of changes in FA scores as a predictor of outcomes in patients with LARC.

In this study, we found that changes in FA scores significantly correlated with oncological outcomes in patients with LARC. We also identified these changes as an independent prognostic parameter for OS and DFS in patients with LARC. The group with increased FA scores after CRT had worse estimated 3-year DFS (63.6% vs. 82.1%) and OS (68.2% vs. 89.3%) compared with the group with decreased or unchanged FA scores after CRT. Patients with increased FA scores had 3.325 times the risk of death compared with those with decreased or unchanged FA scores. This finding remained significant after adjusting for other clinicopathological factors, pointing to increased FA scores as an independent prognostic factor for adverse outcomes. These results can be backed up by clinical and experimental evidence. First, as we mentioned above, plasma Fib levels correlated with tumor progression in colorectal cancer, which indicates that effective treatment may reduce tumor burden and cause reduction in Fib levels. Kawai et al. reported that high post-CRT Fib levels were associated with adverse CRT responses and short disease-free survival and suggested that reductions in plasma Fib levels after NCRT constituted a good biomarker for assessing the efficacy of NCRT [31]. Second, Alb has been reported to have various anticancer properties, such as antioxidant activity, stabilization of DNA replication, and anti-inflammatory effects [32]. Reduced serum Alb after CRT may entail poor nutrition and weak immunity, resulting in increased surgical complications, reduced tolerance of postoperative anticancer therapy, and delayed adjuvant chemotherapy, while every 4 weeks of postponed adjuvant chemotherapy increases mortality by 14% [33]. Moreover, since the combination of Fib and Alb levels is reflection of systemic inflammation and nutritional status, an increase in FA score may point to an organic microenvironment with sustained protumorigenic inflammation and weakened antitumor immunity, conductive to tumor progression, resulting in poor OS and DFS. For these reasons, an increased FA score can be an indicator of adverse survival in patients with LARC.

Furthermore, our study showed that the group with increased FA score contained a higher proportion of patients aged >60 years than the group with decreased or unchanged FA scores. Changes in FA scores were not associated with tumor pathological stage or histological grade; thus, they may reflect host status rather than tumor biology, as increasing age was usually associated with high levels of asymptomatic inflammation and increased secretion of procoagulant and proinflammatory cytokines.

NCRT combined with TME surgery has improved local control greatly, leading to distant metastases as the most prominent source of postoperative failure. Therefore, the preemptive identification of patients at high risk of distant metastases will help clinicians to formulate appropriate treatment protocols and follow-up plans. In this study, a significant difference in DMFS was observed between the two groups, with a 3-year DMFS rate of 67.2% in group B and 84.5% in group A (*P* = 0.022). Increased FA scores could be a sign of systemic recurrence. Thus, especially in the increased FA score group, intensive follow-up and careful assessment of systemic failure should be recommended, and immediate adjuvant chemotherapy after surgery or more aggressive adjuvant chemotherapy should be considered.

Our conclusions may be flawed by the retrospective nature of our study and its relatively small sample size. Moreover, follow-up time was not sufficiently long, with median follow-up shorter than 4 years. Besides, we defined Fib and Alb cut-off values according to our hospital's reference values. Appropriate cut-off values for Fib and Alb levels warrant further exploration. In addition, the variables of Fib and Alb could be influenced by other conditions, such as smoking status and alcohol intake, which were not recorded in this study.

In conclusion, this is the first study reporting that changes in FA score significantly correlates with OS and DFS in patients with rectal cancer, with increased FA scores in patients with worse OS and DFS. Although our findings require further verification, the current study implies that changes in FA score can be used to predict outcomes in NCRT-treated patients with LARC.

## Figures and Tables

**Table 1:** Patient clinicopathologic characteristics.

		Value
Age (years)	Median (range)	60 (19-83)
cTNM	II/III	45/61
Pathology	Adenocarcinoma/mucinous	101/5
ypT	0/1/2/3/4	24/5/46/29/2
ypN	0/1/2	80/18/8
pCR	Yes/no	23/83
ypTNM	0/I/II/III/IV	23/40/17/26/0
CEA	Median (range)	3.20 (0.50-172.92)
CA19-9	Median (range)	11.40 (1.20-1022.50)
Pre-CRT Fib (mg/dl)	Median (range)	3.45 (0.50-6.84)
Post-CRT Fib (mg/dl)	Median (range)	3.37 (1.85-7.67)
Pre-CRT Alb (g/l)	Median (range)	40.35 (27.0-50.20)
Post-CRT Alb (g/l)	Median (range)	40.40 (23.4-51.90)
Pre-CRT FA score	0/1/2	85/16/5
Post-CRT FA score	0/1/2	75/24/7

pCR: pathologic complete response; CEA: carcinoembryonic antigen; CA19-9: carbohydrate antigen 19-9; CRT: chemoradiotherapy; Fib: fibrinogen; Alb: albumin; FA: fibrinogen and albumin.

**Table 2:** Association between clinicopathological factors and changes in FA scores.

	FA score decreased or unchanged (*n* = 84)	FA score increased (*n* = 22)	*P*
Age (years)			0.017
≤60	47	6	
>60	37	16	
Sex			0.219
Female	23	9	
Male	61	13	
Distance from anal verge (cm)			0.436
≤5	46	10	
>5	38	12	
Pathologic differentiation			0.938
Well, moderately	72	19	
Poorly, mucinous	12	3	
CEA (ng/ml)			0.872
≤5	55	14	
>5	29	8	
CA19-9 (ng/ml)			0.112
≤37	70	15	
>37	14	7	
Pathologic T stage			0.766
T0-2	60	15	
T3-4	24	7	
Pathologic N stage			0.825
N0	63	17	
N+	21	5	
LVI			0.073
Negative	74	16	
Positive	10	6	
PNI			0.730
Negative	70	19	
Positive	14	3	
pCR			0.303
Yes	20	3	
No	64	19	
Downstage			0.971
Yes	50	13	
No	34	9	
Pre-CRT Fib	3.38 ± 0.98	3.72 ± 0.63	0.051^∗^
Post-CRT Fib	3.27 ± 0.74	4.33 ± 1.13	≤0.001^∗^
Pre-CRT Alb	40.73 ± 4.45	40.27 ± 3.31	0.649^∗^
Post-CRT Alb	40.92 ± 4.04	36.60 ± 5.75	0.003^∗^

pCR: pathologic complete response; CEA: carcinoembryonic antigen; CA19-9: carbohydrate antigen 19-9; CRT: chemoradiotherapy; LVI: lymph vascular invasion; PNI: perineural invasion; Fib: fibrinogen; Alb: albumin; FA: fibrinogen and albumin. ^∗^Independent *t*-tests were used to analyze with continuous variables.

**Table 3:** Univariate analysis for 3-year DFS and OS rate.

Variables	*N*	3-year DFS	*P*	3-year OS	*P*
Age			0.690		0.167
≤60	53	79.2%		83.0%	
>60	53	77.4%		86.8%	
Sex			0.950		0.647
Female	32	75%		84.4%	
Male	74	79.7%		85.1%	
Distance from anal verge (cm)			0.127		0.569
≤5	56	73.2%		82.1%	
>5	50	84%		88%	
Pathologic differentiation			0.008		0.030
Well and moderately	91	81.3%		87.9%	
Poorly and mucinous	15	60.0%		66.7%	
CEA (ng/ml)			≤0.001		0.001
≤5	69	91.3%		95.7%	
>5	37	54.1%		64.9%	
CA19-9 (ng/ml)			0.013		0.001
≤37	85	83.5%		90.6%	
>37	21	57.1%		61.9%	
Pathologic T stage			0.332		0.931
T0-2	75	81.3%		85.3%	
T3-4	31	71.0%		83.9%	
Pathologic N stage			0.001		0.005
N0	80	85.0%		88.8%	
N+	26	57.7%		73.1%	
LVI			0.536		0.566
Negative	90	78.9%		84.4%	
Positive	16	75.0%		87.5%	
PNI			0.980		0.537
Negative	89	79.8%		84.3%	
Positive	17	70.6%		88.2%	
pCR			0.024		0.033
Yes	23	95.7%		95.7%	
No	83	73.5%		81.9%	
Downstage			0.003		0.022
Yes	63	87.3%		90.5%	
No	43	65.1%		76.7%	
FA score			0.028		0.002
FA score decreased or unchanged	84	82.1%		89.3%	
FA score increased	22	63.6%		68.2%	

CEA: carcinoembryonic antigen; CA19-9: carbohydrate antigen 19-9; LVI: lymph vascular invasion; PNI: perineural invasion; pCR: pathologic complete response; CRT: chemoradiotherapy; FA: fibrinogen and albumin.

**Table 4:** Multivariate analysis for 3-year DFS and OS.

	Disease-free survival			Overall survival		
	HR	95% CI	*P*	HR	95% CI	*P*
Pathologic differentiation (well and moderately vs. poorly and mucinous)	3.922	1.519-10.125	0.005	2.592	0.972-6.9121	0.057
CEA (≤5 ng/ml vs. >5 ng/ml)	5.741	2.407-13.690	≤0.001	3.832	1.646-8.924	0.002
CA19-9 (≤37 ng/ml vs. >37 ng/ml)	1.566	0.704-3.484	0.272	2.022	0.879-4.650	0.098
Pathologic N stage (N0 vs. N+)	2.381	0.817-6.935	0.112	1.989	0.626-6.316	0.243
pCR (yes vs. no)	2.909	0.624-13.561	0.174	3.300	0.720-15.215	0.124
Downstage (yes vs. no)	0.828	0.255-2.690	0.754	0.743	0.217-2.541	0.636
FA score changes (decreased or unchanged vs. increased)	2.433	1.102-5.375	0.028	3.325	1.468-7.532	0.002

CEA: carcinoembryonic antigen; CA19-9: carbohydrate antigen 19-9; pCR: pathologic complete response; FA: fibrinogen and albumin; HR: hazard ratio; CI: confidential interval.

## Data Availability

The clinical data used to support the findings of this study are available from the corresponding author upon request.

## References

[1] Bray F., Ferlay J., Soerjomataram I., Siegel R. L., Torre L. A., Jemal A. (2018). Global cancer statistics 2018: GLOBOCAN estimates of incidence and mortality worldwide for 36 cancers in 185 countries. *CA: a Cancer Journal for Clinicians*.

[2] Arnold M., Sierra M. S., Laversanne M., Soerjomataram I., Jemal A., Bray F. (2017). Global patterns and trends in colorectal cancer incidence and mortality. *Gut*.

[3] Sauer R., Becker H., Hohenberger W. (2004). Preoperative versus postoperative chemoradiotherapy for rectal cancer. *The New England Journal of Medicine*.

[4] Sebag-Montefiore D., Stephens R. J., Steele R. (2009). Preoperative radiotherapy versus selective postoperative chemoradiotherapy in patients with rectal cancer (MRC CR07 and NCIC-CTG C016): a multicentre, randomised trial. *The Lancet*.

[5] Sánchez-Lara K., Turcott J. G., Juárez E. (2012). Association of nutrition parameters including bioelectrical impedance and systemic inflammatory response with quality of life and prognosis in patients with advanced non-small-cell lung cancer: a prospective study. *Nutrition and Cancer*.

[6] Wang J., Liu H., Shao N. (2015). The clinical significance of preoperative plasma fibrinogen level and platelet count in resectable esophageal squamous cell carcinoma. *World Journal of Surgical Oncology*.

[7] Hung H. Y., Chen J. S., Yeh C. Y. (2011). Effect of preoperative neutrophil-lymphocyte ratio on the surgical outcomes of stage II colon cancer patients who do not receive adjuvant chemotherapy. *International Journal of Colorectal Disease*.

[8] Huang J., Wang Y., Yuan Y. (2017). Preoperative serum pre-albumin as an independent prognostic indicator in patients with localized upper tract urothelial carcinoma after radical nephroureterectomy. *Oncotarget*.

[9] Li N., Tian G.-W., Wang Y., Zhang H., Wang Z.-h., Li G. (2017). Prognostic role of the pretreatment C-reactive protein/albumin ratio in solid cancers: a meta-analysis. *Scientific Reports*.

[10] Sun K., Chen S., Xu J., Li G., He Y. (2014). The prognostic significance of the prognostic nutritional index in cancer: a systematic review and meta-analysis. *Journal of Cancer Research and Clinical Oncology*.

[11] Takeuchi H., Ikeuchi S., Kitagawa Y. (2007). Pretreatment plasma fibrinogen level correlates with tumor progression and metastasis in patients with squamous cell carcinoma of the esophagus. *Journal of Gastroenterology and Hepatology*.

[12] Jones J. M., McGonigle N. C., McAnespie M., Cran G. W., Graham A. N. (2006). Plasma fibrinogen and serum C-reactive protein are associated with non-small cell lung cancer. *Lung Cancer*.

[13] Son H. J., Park J. W., Chang H. J. (2013). Preoperative plasma hyperfibrinogenemia is predictive of poor prognosis in patients with nonmetastatic colon cancer. *Annals of Surgical Oncology*.

[14] Lien Y. C., Hsieh C. C., Wu Y. C. (2004). Preoperative serum albumin level is a prognostic indicator for adenocarcinoma of the gastric cardia. *Journal of Gastrointestinal Surgery*.

[15] González-Trejo S., Carrillo J. F., Carmona-Herrera D. D. (2017). Baseline serum albumin and other common clinical markers are prognostic factors in colorectal carcinoma. *Medicine*.

[16] Zhang Y., Xiao G. (2019). Prognostic significance of the ratio of fibrinogen and albumin in human malignancies: a meta-analysis. *Cancer Management and Research*.

[17] Matsuda S., Takeuchi H., Kawakubo H. (2015). Cumulative prognostic scores based on plasma fibrinogen and serum albumin levels in esophageal cancer patients treated with transthoracic esophagectomy: comparison with the Glasgow prognostic score. *Annals of Surgical Oncology*.

[18] Wang Y., Chen W., Hu C. (2017). Albumin and fibrinogen combined prognostic grade predicts prognosis of patients with prostate cancer. *Journal of Cancer*.

[19] Collen D., Tytgat G. N., Claeys H., Piessens R. (1972). Metabolism and distribution of fibrinogen. I. Fibrinogen turnover in physiological conditions in humans. *British Journal of Haematology*.

[20] Zhang Z., Fuller G. M. (2000). Interleukin 1beta inhibits interleukin 6-mediated rat gamma fibrinogen gene expression. *Blood*.

[21] Perisanidis C., Psyrri A., Cohen E. E. (2015). Prognostic role of pretreatment plasma fibrinogen in patients with solid tumors: a systematic review and meta-analysis. *Cancer Treatment Reviews*.

[22] Man Y. N., Wang Y. N., Hao J. (2015). Pretreatment plasma D-dimer, fibrinogen, and platelet levels significantly impact prognosis in patients with epithelial ovarian cancer independently of venous thromboembolism. *International Journal of Gynecological Cancer*.

[23] Qi Q., Geng Y., Sun M., Chen H., Wang P., Chen Z. (2015). Hyperfibrinogen is associated with the systemic inflammatory response and predicts poor prognosis in advanced pancreatic cancer. *Pancreas*.

[24] Tang L., Liu K., Wang J., Wang C., Zhao P., Liu J. (2010). High preoperative plasma fibrinogen levels are associated with distant metastases and impaired prognosis after curative resection in patients with colorectal cancer. *Journal of Surgical Oncology*.

[25] Martino M. M., Briquez P. S., Ranga A., Lutolf M. P., Hubbell J. A. (2013). Heparin-binding domain of fibrin(ogen) binds growth factors and promotes tissue repair when incorporated within a synthetic matrix. *Proceedings of the National Academy of Sciences of the United States of America*.

[26] Seebacher V., Polterauer S., Grimm C. (2010). The prognostic value of plasma fibrinogen levels in patients with endometrial cancer: a multi-centre trial. *British Journal of Cancer*.

[27] McMillan D. C., Watson W. S., O'Gorman P., Preston T., Scott H. R., McArdle C. (2001). Albumin concentrations are primarily determined by the body cell mass and the systemic inflammatory response in cancer patients with weight loss. *Nutrition and Cancer*.

[28] Brenner D. A., Buck M., Feitelberg S. P., Chojkier M. (1990). Tumor necrosis factor-alpha inhibits albumin gene expression in a murine model of cachexia. *The Journal of Clinical Investigation*.

[29] Cengiz O., Kocer B., Sürmeli S., Santicky M. J., Soran A. (2006). Are pretreatment serum albumin and cholesterol levels prognostic tools in patients with colorectal carcinoma?. *Medical Science Monitor*.

[30] Heys S. D., Walker L. G., Deehan D. J., Eremin O. E. (1998). Serum albumin: a prognostic indicator in patients with colorectal cancer. *Journal of the Royal College of Surgeons of Edinburgh*.

[31] Kawai K., Kitayama J., Tsuno N. H., Sunami E., Nagawa H. (2011). Hyperfibrinogenemia after preoperative chemoradiotherapy predicts poor response and poor prognosis in rectal cancer. *International Journal of Colorectal Disease*.

[32] Seaton K. (2001). Albumin concentration controls cancer. *Journal of the National Medical Association*.

[33] Biagi J. J., Raphael M. J., Mackillop W. J., Kong W., King W. D., Booth C. M. (2011). Association between time to initiation of adjuvant chemotherapy and survival in colorectal cancer: a systematic review and meta-analysis. *Journal of the American Medical Association*.

